# Triglyceride‐Glucose Index Association With Severity of Coronary Artery Disease

**DOI:** 10.1002/edm2.70025

**Published:** 2025-03-14

**Authors:** Sara Saffar Soflaei, Pooria Salehi‐Sangani, Zahra Fallahi, Fatemeh Imanparast, Mahdieh Marousi, Mohammad Tajfard, Gordon A. Ferns, Mohsen Moohebati, Majid Ghayour‐Mobarhan

**Affiliations:** ^1^ Metabolic Syndrome Research Center Mashhad University of Medical Sciences Mashhad Iran; ^2^ Faculty of Medicine Mashhad University of Medical Sciences Mashhad Iran; ^3^ School of Nursing and Midwifery Mashhad University of Medical Sciences Mashhad Iran; ^4^ School of Medicine North Khorasan University of Medical Sciences Bojnurd Iran; ^5^ Faculty of Medicine Islamic Azad University Medical Branch of Mashhad Mashhad Iran; ^6^ Department of Health Education and Health, Faculty of Health Promotion Mashhad University of Medical Sciences Mashhad Iran; ^7^ Brighton and Sussex Medical School Division of Medical Education Brighton UK; ^8^ Heart and Vascular Research Center Mashhad University of Medical Sciences Mashhad Iran; ^9^ Department of Cardiology, Faculty of Medicine Mashhad University of Medical Sciences Mashhad Iran; ^10^ International UNESCO Center for Health‐Related Basic Sciences and Human Nutrition Mashhad University of Medical Sciences Mashhad University of Medical Sciences Mashhad Iran

**Keywords:** angiography, coronary artery disease, insulin resistance, triglyceride–glucose index

## Abstract

**Background:**

Despite previous studies, the relationship between the triglyceride‐glucose (TyG) index and coronary artery disease (CAD) is still undetermined. So we aimed to investigate the association between the TyG index and CAD.

**Method:**

A total of 2346 subjects were enrolled in the study and were categorised into 5: those with no CAD, angiogram negative (Ang−) patients, those with single‐vessel disease (SVD), or two‐vessel disease (2VD) or three‐vessel disease (3VD). Demographic characteristics, disease history and biochemical investigations were recorded. TyG index was calculated as Ln [fasting TG (mg/dL) × fasting glucose (mg/dL)/2].

**Results:**

Adjusted regression models demonstrated that the odds of 3VD (OR, 5.847; 95% CI, 4.391–7.784), 2VD (OR, 4.943; 95% CI, 3.597–6.791), SVD (OR, 4.722; 95% CI 3.448–6.647) and a negative coronary angiogram (OR, 3.137; 95% CI, 2.431–4.049), increased significantly per each unit elevation of the TyG index, compared to the healthy participants. Also, the odds for being 3VD (1.864, 95%CI 1.402–2.477, *p*‐value < 0.001), 2VD (1.575, 95%CI 1.143–2.171, *p*‐value = 0.005) and SVD (1.505, 95%CI 1.097–2.065, *p*‐value = 0.011) were increased significantly by one‐unit elevation of TyG index, compared to Ang− group.

**Conclusion:**

Our study demonstrates a significant association between elevated TyG index and the presence and severity of CAD. Higher TyG index values were consistently linked to an increased likelihood of multivessel CAD, especially in diabetic patients. These findings suggest that the TyG index could serve as a valuable marker for assessing CAD risk and stratification.

## Graphical abstract

Triglyceride‐glucose (TyG) index is associated with the presence of coronary stenosis. The odds of coronary stenosis severity increase by increasing TyG.

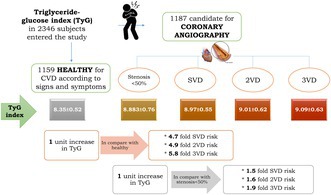

## 1 Introduction

Coronary artery disease (CAD) occurs when an atherosclerotic plaque forms within the coronary arteries and causes decreased blood flow to the myocardium. CAD is one of the significant reasons for impaired general health [1, 2]. Atherosclerotic plaque formation, as a main aetiology that underlies the pathophysiologic mechanisms of CAD, is caused by the incorporation of several factors [3]. Essential roles of some risk factors have been well‐established in large epidemiological studies, which include smoking [4], diabetes and hyperglycemia [5], hyperlipidemia [6], hypertension (HTN) [7], etc. The lipid content, as the main structure of an atherosclerotic plaque, may trigger this process by precipitation and, afterward, oxidation in vascular walls and consequently, endothelial dysfunction [8]. In line with this finding, several investigations implied the significance of the involvement of triglycerides (TGs) and fatty acids in this pathophysiological process [9, 10].

Insulin resistance (IR) is characterised by the impaired capability of insulin to effectively control glucose metabolism. The role of IR in atherosclerotic progression through aberrant lipid metabolism has been shown in several studies [11, 12]. Ultimately, it is evident that IR might have an association with major cardiovascular events, such as CAD [13, 14, 15]. The intravenous glucose tolerance and euglycemic insulin clamp have been established as gold standards for evaluating IR, but due to invasiveness and expensiveness, they are not applicable in the clinical practice [16]. In response to these constraints, the triglyceride‐glucose (TyG) index was developed as a cost‐effective and readily accessible method in 2008, which is calculated as the natural logarithm of [fasting TG (mg/dL) × fasting glucose (mg/dL)/2] [17].

Previous investigations revealed that the TyG index has a strong association and might predict cardiovascular events, such as cerebrovascular diseases, CAD and cervical vascular function [18]. In a meta‐analysis by Akbar et al. [19], a relationship between TyG index and major cardiovascular events in acute coronary syndrome (ACS) patients was evaluated and, their results showed a dose–response relation. In another systematic review and meta‐analysis study, the authors reported that the TyG index might associated with cerebrovascular diseases, however, their study compromised some limitations that necessities more investigations [20]. Moreover, in our previous study, we found that the TyG index, but not TG/HDL‐C, was an independent predictor of coronary stenosis [21]. However, there is no study to examine the association between the TyG index and the number of involved arteries.

CAD is a significant public health concern that places a substantial burden on global morbidity and mortality rates [20]. Assessing IR as a predisposing condition for the development of atherosclerosis may indicate the pre‐CAD condition. The TyG index, a highly reliable method for evaluating IR [22], has shown promising correlations with CAD. However, previous studies have yielded inconclusive results and warrant further investigation in a larger population. Moreover, due to the significant impact of diabetes presence, it is crucial to evaluate the TyG index between diabetic and non‐diabetic patients. Currently, an absence of study examining the potential relation between the CAD and TyG index in diabetic and non‐diabetic populations in our region, specifically concerning the number of affected coronary arteries, is palpated. Herein, we decided to evaluate this hypothesis through a large‐scale population‐based study conducted in the second most populous city in Iran.

## 2 Method

### 2.1 Data Collection

Subjects were recruited from the Ghaem Hospital, Mashhad, the second biggest city in Iran previously explained [23]. A total of 1187 participants were candidates for coronary angiography for the first time, due to chest pain or other symptoms including dyspnea on exertion, between September 2011 and May 2013. In parallel, 1159 healthy controls were randomly selected among those referred to the Ghaem clinic for routine check‐ups. The controls did not have any signs or symptoms of CAD, as established by a cardiologist. Data on the demographic characteristics and medical histories of the population, including age, sex, physical activity level (PAL), smoking, HTN, systolic blood pressure (SBP), diastolic blood pressure (DBP), diabetes mellitus (DM), dyslipidemia (DLP) and, also, biochemical investigations, consisted of fasting blood glucose (FBG), TG, total cholesterol, low‐density lipoprotein (LDL), High‐density lipoprotein (HDL) and high‐sensitivity C‐reactive protein (hs‐CRP). TyG index was calculated using the natural logarithm of fasting TG (mg/dL) multiplied by fasting glucose (mg/dL) and divided by two.

HTN was defined as SBP ≥ 140 mmHg or DBP ≥ 90 mmHg or positive HTN history. DM was defined as FBG ≥ 126 mg/dL or a history of DM. DLP was defined as TG ≥ 150 mg/dL or LDL ≥ 130 mg/dL or HDL < 40 mg/dL in men or HDL < 50 mg/dL in women.

## 3 Angiographic Procedure

Two cardiologists who were unaware of the hs‐CRP level and the other biochemical results evaluated all the angiograms. The severity of coronary obstruction was determined by calculating the total percent diameter stenosis in standard index units (50% = 0.50) [24]. Those who had coronary obstructions were classified into four categories: SVD (one‐stenosis vessel), 2VD (two‐stenosis vessel), 3VD (three‐stenosis vessel), and those who had potential symptoms or signs representing CAD, but had < 50% obstruction and were classified as angiogram negative (Ang−).

## 4 Statistical Analysis

SPSS software version 25.0 was utilised to perform analyses. The data for continuous and categorical variables were presented as mean values with standard deviation and percentages, respectively. To test the distribution of continuous variables, Kolmogrov–Smirnov was utilised. Continuous variables that did not show normal distribution, transformed logarithmically. One‐way analysis of variance (ANOVA) test, unpaired 2‐tailed *t*‐test, Mann–Whitney *U*‐test and the chi‐squared test were utilised to evaluate the differences among the cohorts multinomial logistic regression was used to analyse the feasible relationship between the TyG index and angiographic results. A *p*‐value below 0.05 was considered statistically significant.

## 5 Results

The 2346 participants involved in our study, were divided into five categorical cohorts: (1) healthy and (2) based on the coronary angiography results were categorised into Ang−, SVD, 2VD and 3VD. Table 1 displays the demographic and clinical traits of the study participants. Among all patients included in our study, the mean age was 61.8 ± 8.5 years and 53.08% (*n* = 1194) were male. As shown in Table 1, the average age in the healthy group compared with every patient group who had different degrees of stenosis was substantially lower (*p*‐value < 0.001). In the comparison of the healthy participants with other groups, SBP and DBP had a remarkable increase in the healthy cohort. Unexpectedly smoking rate was higher in the healthy group and does not have any significant differences in any comparison. The proportions of cardio‐metabolic risk factors, including DM, DLP and HTN were dramatically higher in the 3VD group than in the others (*p*‐value < 0.01). Body mass index (BMI) was not significantly different between the cohorts (*p*‐value < 0.461).

**TABLE 1:** Features of the five study groups.

Variables	Healthy (*n* = 1159)	Ang− (*n* = 406)	SVD (*n* = 211)	2VD (*n* = 215)	3VD (*n* = 335)	*p*
Age^a^ (years)	52.97 ± 9.35	53.69 ± 11.43	56.27 ± 11.08	85.40 ± 10.17	60.75 ± 10.20	< 0.001^2,3,4,5,6,7,9^
Men, *n* (%)	584 (50.4)	130 (32.0)	121 (57.3)	134 (62.3)	225 (63.4)	< 0.001
BMI^a^ (kg/m^2^)	26.77 ± 4.19	26.89 ± 5.15	26.99 ± 4.67	27.40 ± 5.66	27.04 ± 5.22	0.461
PAL^a^	1.43 ± 0.26	1.43 ± 0.22	1.42 ± 0.21	1.42 ± 0.22	1.41 ± 0.22	0.546
Smoking, *n* (%)	230 (19.8)	62 (15.3)	53 (25.1)	60 (27.9)	76 (21.4)	< 0.001
DM history, *n* (%)	22 (5.4)	111 (27.1)	67 (16.3)	70 (17.1)	140 (34.1)	< 0.001
HTN history, *n* (%)	85 (13.5)	174 (27.7)	94 (15.0)	89 (14.2)	186 (29.6)	< 0.001
DLP history, *n* (%)	160 (26.4)	137 (22.6)	75 (12.4)	91 (15.0)	142 (23.5)	< 0.001
Metformin consumption, *n* (%)	4 (3.7)	28 (26.2)	27 (25.2)	15 (14.1)	33 (30.8)	< 0.001
SBP^a^ (mmHg)	120.61 ± 15.49	129.75 ± 23.73	132.67 ± 24.00	136.35 ± 25.48	134.48 ± 25.28	< 0.001^1,2,3,4,6,7^
DBP^a^ (mmHg)	74.88 ± 9.90	79.94 ± 11.97	81.14 ± 10.93	82.36 ± 10.60	82.15 ± 11.26	< 0.001^1,2,3,4,7^

*Note:* 1Healthy vs. Ang−/^2^healthy vs. SVD/^3^healthy vs. 2VD/^4^healthy vs. 3VD/^5^Ang− vs. SVD/^6^Ang− vs. 2VD/^7^Ang− vs. 3VD/^8^SVD vs. 2VD/^9^SVD vs. 3VD/^10^2VD vs. 3VD.

Abbreviations: BMI, Body mass index; DBP, diastolic blood pressure; DLP, Dyslipidemia; DM, Diabetes mellitus; HTN, hypertension; PAL, physical activity level; SBP, systolic blood pressure.

^a^ Expressed as mean ± SD.

Table 2 presents the experimental data on biochemical markers in different study groups. FBG and TG were measured in the case and control groups, and the difference between the healthy and angiogram‐negative groups was significant (*p*‐value < 0.001).

**TABLE 2:** Biochemical markers in different study groups.

Variables	Healthy *n* = (1159)	Ang− *n* = (406)	SVD *n* = (211)	2VD *n* = (215)	3VD *n* = (335)	*p*
FBG^b^ (mg/dL)	83.95 ± 17.77	115.74 ± 46.77	124.89 ± 47.16	127.48 ± 56.29	141.47 ± 72.64	< 0.001^1,2,3,4,6,7,9,10^
Total^b^ Cholesterol (mg/dL)	182.8 ± 34.34	168.15 ± 43.77	165.77 ± 41.57	170.36 ± 39.54	170.18 ± 45.86	< 0.001^1,2,3,4^
TG^a^ (mg/dL)	101.00 (74.00–138.00	132.00 (95.00–152.25)	143.00 (102.00–164.00)	144.00 (102.00–167.00)	142.00 (108.00–161.00)	< 0.001^1,2,3,4^
HDL^b^ (mg/dL)	44.47 ± 9.54	42.48 ± 11.60	42.33 ± 17.10	40.36 ± 9.48	40.87 ± 16.99	< 0.001^1,3,4^
LDL^b^ (mg/dL)	113.27 ± 30.79	97.70 ± 35.20	92.82 ± 31.99	101.55 ± 40.41	99.42 ± 31.99	< 0.001^1,2,3,4,8^
hs‐CRP^a^ (mg/dL)	1.30 (0.88–1.79)	4.35 (1.98–5.19)	5.62 (2.34–6.53)	5.63 (2.17–6.78)	5.83 (2.70–6.63)	< 0.001^1,2,3,4,6,7^

*Note:* 1Healthy vs. Ang−/^2^healthy vs. SVD/^3^healthy vs. 2VD/^4^healthy vs. 3VD/^5^Ang− vs. SVD/^6^Ang− vs. 2VD/^7^Ang− vs. 3VD/^8^SVD vs. 2VD/^9^SVD vs. 3VD/^10^2VD vs. 3VD.

Abbreviations: FBG, fasting blood glucose; HDL, high‐density lipid; hs‐CRP, high‐sensitivity C‐reactive protein; LDL, low‐density lipid; TG, triglyceride; TyG, triglyceride and glucose index.

^a^ Expressed as mean ± SD.

^b^ Expressed as median (Q1_Q3).

Patients in the Ang− group had substantially lower levels of total cholesterol, serum LDL and HDL concentrations than the healthy subjects (*p*‐value < 0.001).

A closer inspection of the table shows the total cholesterol, LDL and TG index among 2VD subjects were superior to those in the 3VD group. What stands out in this table is the high rate of hs‐CRP, and the TyG index demonstrated a statistically significant elevation in the 3VD group in comparison to the other groups (*p*‐value < 0.001).

**FIGURE 1:**
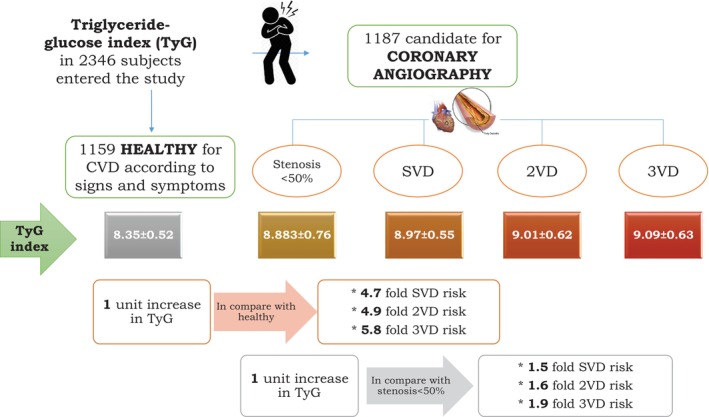
Summary of methodology and results of the study.

As shown in Table 3, after adjustment for gender, age, smoking and individual history of HTN, DM and dyslipidemia, in model 1, one‐unit increase in TyG index was dramatically related to 3.14 (95% CI 2.43–4.05), 4.72 (95% CI 3.45–6.65), 4.94 (95% CI 3.60–6.79) and 5.85 (95% CI 4.39–7.78) fold elevated odds of Ang−, SVD, 2VD and, 3VD in comparison to healthy group, respectively (All *p*‐values < 0.001). Also, regarding model 2, the odds for being 3VD (1.864, 95% CI 1.402–2.477, *p*‐value < 0.001), 2VD (1.575, 95% CI 1.143–2.171, *p*‐value = 0.005) and SVD (1.505, 95% CI 1.097–2.065, *p*‐value = 0.011) was increased significantly per each unit elevation of TyG index, compared to Ang− group. However, regarding model three, by TyG index increment, the odds of being 3VD (1.238, CI 95% 0.904–1.696, *p*‐value = 0.183) and 2VD (1.047, CI 95% 0.737–1.487, *p*‐value = 0.799) in comparison to SVD, was not significant (summarised in Figure 1).

**TABLE 3:** Association of TyG and angiography results.

	Crude	Partial adjusted^a^	Fully adjusted^b^
	OR (CI 95%)	OR (CI 95%)	OR (CI 95%)
Model 1*			
Healthy	Ref.	Ref.	Ref.
Ang−	5.06 (4.04–6.33)**	4.85 (3.87–6.08)**	3.14 (2.43–4.05)**
SVD	7.66 (5.80–10.13)**	7.52 (5.68–9.97)**	4.72 (3.45–6.65)**
2VD	8.38 (6.35–11.06)**	8.20 (6.18–10.89)**	4.94 (3.60–6.79)**
3VD	10.32 (8.07–13.20)**	10.05 (7.79–12.97)**	5.85 (4.39–7.78)**
Model 2			
Ang−	Ref.	Ref.	Ref.
SVD	1.52 (1.14–2.03)*	1.67 (1.40–2.25)**	1.51 (1.097–2.07)*
2VD	1.67 (1.26–2.22**	1.88 (1.39–2.54))**	1.58 (1.143–2.17)*
3VD	2.07 (1.61–2.65)**	2.38 (1.82–3.12)**	1.86 (1.40–2.48)**
Model 3			
SVD	Ref.	Ref.	Ref.
2VD	1.10 (0.80–1.50)	1.14 (0.83–1.58)	1.05 (0.74–1.49)
3VD	1.36 (1.02–1.81)*	1.47 (1.09–1.97)*	1.24 (0.90–1.70)

Abbreviations: OR, odd ratio; Ref., reference.

^a^ Adjusted for age and gender.

^b^ Adjusted for age, gender, smoking and individual history of HTN, DM and DLP.

* *p‐*value < 0.05.

** *p‐*value < 0.001.

As shown in Table 4, among individuals with no history of DM, the TyG index increased progressively from healthy individuals (8.34 ± 0.50) to those with CAD across the Angina, SVD (8.86 ± 0.48), 2VD (8.82 ± 0.49) and 3VD (8.85 ± 0.50) groups (*p* < 0.001). In individuals with a history of DM, the TyG index followed a similar trend, with higher TyG values observed in patients with multivessel disease (SVD: 9.22 ± 0.62, 2VD: 9.38 ± 0.69, 3VD: 9.45 ± 0.64) compared to healthy individuals (9.21 ± 0.76). A statistically significant difference was observed between the Angina group and the 3VD group among diabetic patients (*p* = 0.007).

**TABLE 4:** TyG descriptive in different groups of coronary stenosis according to the history of DM.

	History of DM−			History of DM+		
	No	TyG (mean ± SD)	*p*	No	TyG (mean ± SD)	*p*
Healthy	1137	8.34 ± 0.50	< 0.001^1,2,3,4,5,6^	22	9.21 ± 0.76	0.007^6^
Ang−	295	8.70 ± 0.50		111	9.17 ± 0.61	
SVD	144	8.86 ± 0.48		67	9.22 ± 0.62	
2VD	145	8.82 ± 0.49		70	9.38 ± 0.69	
3VD	215	8.85 ± 0.50		140	9.45 ± 0.64	
Total	1936	8.52 ± 0.55		410	9.31 ± 0.66	

*Note:* ^1^Healthy vs. Ang−/^2^healthy vs. SVD/^3^healthy vs. 2VD/^4^healthy vs. 3VD/^5^Ang− vs. SVD/^6^Ang− vs 3VD.

Abbreviations: DM, diabetes mellitus; TyG, triglyceride and glucose index.

As shown in Table 5, after adjustment for gender, age, smoking and individual history of HTN, DM and dyslipidemia, among non‐diabetic patients, one‐unit increase in TyG index was dramatically related to 3.41 (95% CI 2.55–4.57), 6.63 (95% CI 4.47–9.86), 5.40(95% CI 3.75–8.19) and 6.08 (95% CI 4.29–8.63) fold elevated odds of Ang−, SVD, 2VD and 3VD in comparison to healthy group, respectively (All *p*‐values < 0.001). Also, regarding model 2, the odds for being 3VD (1.98, 95%CI 1.32–2.97, *p*‐value < 0.001), 2VD (1.74, 95%CI 1.12–2.69, *p*‐value < 0.05) and SVD (1.99, 95%CI 1.29–3.10, *p*‐value < 0.05) was increased significantly per each unit elevation of TyG index, compared to Ang‐ group. However, regarding model three, by TyG index increment, the odds of being 3VD (1.01, CI 95% 0.64–1.58, *p*‐value > 0.05) and 2VD (0.86, CI 95% 0.53–1.39, *p*‐value > 0.05) in comparison to SVD, was not significant.

**TABLE 5:** Association of TyG and angiography results in participants without DM.

	Crude	Partial adjusted^a^	Fully adjusted^b^
	OR (95%CI)	OR (95%CI)	OR (95%CI)
Model 1			
Healthy	Ref.	Ref.	Ref.
Ang−	5.19 (3.20–5.48)**	4.18 (3.18–5.48)**	3.41 (2.55–4.57)**
SVD	7.86 (5.43–11.37)**	7.64 (5.26–11.10)**	6.63 (4.47–9.83)**
2VD	6.82 (4.74–9.83)**	6.45 (4.45–9.35)**	5.40 (3.75–8.19)**
3VD	7.49 (5.45–10.28)**	6.92 (4.98–9.60)**	6.08 (4.29–8.63)**
Model 2			
Ang−	Ref.	Ref.	Ref.
SVD	1.91 (1.27–2.89)*	2.05 (1.34–3.15)**	1.99 (1.29–3.10)*
2VD	1.65 (1.10–2.48)*	1.82 (1.19–2.79)*	1.74 (1.12–2.69)*
3VD	1.82 (1.27–2.61)**	2.07 (1.40–3.07)**	1.98 (1.32–2.97)**
Model 3			
SVD	Ref.	Ref.	Ref.
2VD	0.86 (0.54–1.38)	0.89 (0.55–1.43)	0.86 (0.53–1.39)
3VD	0.95 (0.62–1.47)	1.01 (0.65–1.57)	1.01 (0.64–1.58)

Abbreviations: OR, odd ratio; Ref., reference.

^a^ Adjusted for age and gender.

^b^ Adjusted for age, gender, smoking, and individual history of HTN, DM and DLP.

* *p*‐value < 0.05.

** *p*‐value < 0.001.

Table 6 indicates the odds of TyG level for coronary stenosis severity in diabetic participants. TyG levels were not associated with coronary stenosis in the first model both crude and adjusted models (*p* > 0.05). In the second model as considering Ang− for reference 1 unit increase in TyG increases the ORs of 2VD and 3VD by 1.87 and 2.26 folds respectively after adjusting for all confounding factors. Moreover, a 1 unit increase in TyG increases the risk of 3VD by 2.02 (CI 95%:1.23–3.32) after adjustment compared with SVD.

**TABLE 6:** Association of TyG and angiography results in participants with DM.

	Crude	Partially adjusted^a^	Fully adjusted^b^
	OR (95%CI)	OR (95%CI)	OR (95%CI)
Model 1			
Healthy	Ref.		—
Ang−	0.91 (0.44–1.88)	0.85 (0.40–1.79)	0.85 (0.39–1.86)
SVD	1.01 (0.47–2.18)	1.025 (0.47–2.23)	1.01 (0.45–2.28)
2VD	1.51 (0.71–3.23)	1.60 (0.74–3.43)	1.56 (0.55–4.39)
3VD	1.77 (0.68–3.63)	1.91 (0.92–3.94)	1.88 (0.88–4.02)
Model 2			
Ang−	Ref.	Ref.	Ref.
SVD	1.12 (0.6901.84)	1.20 (0.73–1.99)	1.20 (0.72–2.01)
2VD	1.69 (1.05–2.73)*	1.88 (1.14–3.11)*	1.87 (1.12–3.11)*
3VD	1.99 (1.33–2.99)**	1.26 (1.45–3.53)**	2.26 (1.44–3.55)**
Model 3			
SVD	Ref.	Ref.	Ref.
2VD	1.50 (0.88–2.55)	1.64 (0.95–2.83)	1.63 (0.94–2.83)
3VD	1.77 (1.11–2.82)*	2.02 (1.23–3.31)*	2.02 (1.23–3.32)*

Abbreviations: OR, odd ratio; Ref., reference.

^a^ Adjusted for age and gender.

^b^ Adjusted for age, gender, smoking, and individual history of HTN, DM, and DLP.

* *p*‐value < 0.05.

** *p*‐value < 0.001.

## 6 Discussion

The findings of our study have shown that the serum TyG index and angiographically‐defined CAD are associated. In addition, we revealed that a greater value of the TyG index could represent multivessel involvement of coronary arteries. As we pointed out, in our study population, CAD was related to a higher TyG index. Additionally, we elucidated the capacity of the TyG index to discern individuals with negative angiographic findings from those with positive angiographic results. Furthermore, through the use of multinomial logistic regression, our research suggests that a one‐unit enhancement of the serum TyG index is related to a notable increase greater than 3, 4.7, 4.9, a 5.8‐fold increase in the odds of presenting with Ang−, SVD, 2VD and 3VD compared to healthy individuals, respectively. Additionally, involvement of 3, 2 and 1 coronary vessel, was correlated with about 1.9, 1.6 and 1.5 fold odds of higher TyG index in comparison to angiogram negative patients. To the extent of our current understanding, the results of previous studies, necessitate more studies to appraise the feasible association between the CAD and TyG index, thus we evaluated the relationship between the TyG index and CAD defined by angiography. Moreover, TyG could be helpful in the diagnosis of coronary stenosis specifically in diabetic patients.

The TyG index is defined as the natural logarithm of [fasting TG (mg/dL) × fasting glucose (mg/dL)/2]. Previous studies have indicated that one possible proxy indicator of IR is the serum TyG index [25, 26]. IR refers to a condition in which, insulin cannot affect as it is expected. IR could cause endothelial cell dysfunction through lower nitric oxide production, which finally leads to more vasoconstriction. Furthermore, IR affects vascular smooth muscle cell proliferation, and pro‐inflammatory substance secretion [27, 28]. Insulin regulates the production of very low‐density lipoprotein (VLDL) to limit the level of plasma TGs [29, 30], and consequently, the aberrant insulin effect leads to hypertriglyceridemia. Studies have demonstrated the undeniable involvement of elevated levels of VLDL and TGs in the pathophysiology of atherosclerosis [31]. Thus, these findings brought up the assumption that IR and atherosclerosis, as a major risk factor for CAD, have a strong significant association which has also been approved in several antecedent studies [32, 33, 34, 35].

Prior research has demonstrated that IR predicts cardiovascular events. A previous study showed that IR is significantly more prevalent in patients with HTN which is one of the traditional risk factors for CAD [36]. In a cohort of 2938 individuals, IR was brought up as a significant risk factor for CAD [37]. In a systematic review and meta‐analysis that comprised 516,325 subjects, IR was evaluated using the HOMA index, and the results demonstrated that IR may emerge as a promising independent factor in predicting CAD [38]. Additionally, another study revealed that IR is related to an elevated risk of all‐cause mortality and cardiovascular events [39].

The TyG index has been widely recognised as a dependable measure for IR [22]. Previous research have shown the usage of TyG index in assessing several diseases. For instance a previous meta‐analysis showed that TyG index as a potential risk stratification value in ischemic stroke patients [40]. Similarly another study revealed that TyG index could be used to assess the risk of heart failure incidence in different populations [41]. Moreover, in addition to risk assessment, it has been found that TyG index might be insightful in management of CAD patients [42, 43]. In resemblance with our findings, other studies revealed that the TyG index identifies individuals with a high risk of CAD and arterial stiffness [44, 45]. In a study by Wang and colleagues, their results showed a strong risk of multivessel CAD and a higher level of TyG index, specifically in patients with pre‐DM conditions [46]. They recruited 2792 participants with an average age of 66, and most were male findings indicated that, following age and gender adjustment, the TyG index, when considered as a discrete variable, could serve as a predictor of multivessel CAD with an odds ratio of 1.4, which is also consistence with our study. However, they did not categorise multivessel CAD patients based on the number of involved arteries. Similarly, a large cohort study, revealed that the TyG index estimates the incidence of cardiovascular events [47]. The patients were classified into four groups regarding their TyG index, and individuals in the fourth, third and second quartiles exhibited hazard ratios of 1.3, 1.2 and 1.1 respectively, concerning the first quartile as a reference, for the occurrence of myocardial infarction. In addition, Mahdavi‐Roshan et al. recruited 3085 and 774 subjects as non‐CAD and CAD participants. Patients were diagnosed by angiography‐based method. Their results revealed that the TyG index one‐unit elevation was associated to about 4‐fold greater CAD risk [48].

Wang et al. [49] implemented the same hypothesis on a group of 935 patients with the presentation of ACS. They divided patients into TyG index quartiles (1–4 groups). Their study revealed that the TyG index might emerge as a highly associated index with coronary disease. In line with our observation, they found that, by each unit elevation in the TyG index, the chance of occurring multivessel coronary disease elevated by about 2.2. Also, they divided patients into three groups according to the number of involved coronary arteries (less or one involved artery, two involved arteries, and three or more involved arteries). They found that the TyG index, as a quartile stratification variable, significantly predicted the severity of the disease. However in our study, according to model 3, the TyG index as a discrete variable could not differentiate patients based on the numbers of the involved arteries. One possible reason for this controversy is the difference between the assuming TyG index as a discrete or categorical variable. Another reason that might underlie this controversy is the setting of subjects. It is claimable that, the TyG index in participants with ACS may play a stronger role in predicting the numbers of involved arteries, whilst in our study population, patients were not presented with ACS necessarily. In contrast with the study of Wang et al. [50], in another study, 2531 patients with ACS and DM were recruited. According to their findings, the TyG index does not predict nonfatal myocardial ischemia, nonfatal stroke and all‐cause disease. According to model 3, this finding is consistence with our finding, and together, might demonstrate that the TyG index is not capable of predicting prognosis among patients with more than 50% obstruction. In summary, more studies are needed to determine the prediction significance of the TyG index among participants in acute settings of coronary diseases to differentiate them by the severity of CAD.

The findings from our study demonstrate a clear association between the TyG index and the progression of CAD in both diabetic and non‐diabetic individuals. Specifically, our data show a progressive increase in the TyG index from healthy individuals to those with CAD, and further increases with the severity of vessel involvement. This pattern was consistently observed in individuals both with and without DM, highlighting the robustness of the TyG index as a potential marker for CAD severity. Our results align with previous research indicating that the TyG index is elevated in individuals with CAD and is associated with increased cardiovascular risk. In studies conducted on non‐diabetic populations, similar trends have been noted, where individuals with CAD had higher TyG values compared to healthy individuals, with a gradual increase in TyG values correlating with the number of affected vessels (2VD and 3VD). For instance, in a study by Park et al. [51] the TyG index was shown to be a reliable predictor of coronary artery calcification, with the index significantly higher in multivessel disease cases compared to single‐vessel disease, mirroring our findings.

Among diabetic individuals, our findings of elevated TyG index in multivessel disease (SVD, 2VD and 3VD) are consistent with studies that have established a heightened risk of CAD in diabetic populations with elevated TyG levels. Prior research has similarly demonstrated that diabetic patients tend to have higher baseline TyG indices, with even greater elevations observed in those with more extensive vessel involvement. For example, a study by Lee et al. (2020) observed a higher TyG index is linked to an elevated risk of CAS in asymptomatic individuals with type 2 diabetes, especially among those with additional cardiovascular risk factors [52].

This study has several strengths. First of all, huge numbers of individuals were utilised and consequently, the results are strongly trustworthy. Secondly, the evaluation of the series characteristics of participants, empowered us for further model construction to appraise the true and adjusted relationship between the TyG index and the prevalence of CAD. However, some factors could affect and limit our study. Firstly, it is mentionable that, this study was only conducted in one city, however, it is the second big populated city in Iran. Secondly, lack of prospective follow‐up data might constrain our results. Moreover, our data did not contain the HbA1c data of patients, which should be considered in future studies.

TyG index can serve as a useful and reliable marker for identifying individuals at higher risk of CAD, particularly multivessel disease. Elevated TyG index values are significantly associated with the presence and severity of CAD, making it a valuable tool for risk stratification in clinical practice. Its ability to distinguish between patients with and without angiographically confirmed CAD suggests that it could aid in the early detection and management of CAD, especially in populations with IR or metabolic abnormalities.

## 7 Conclusion

Our study has shown a strong association between the TyG index and the presence of CAD. Additionally, our study demonstrated a correlation between increased TyG index levels and an elevated likelihood of multivessel CAD especially in diabetic patients.

## Author Contributions

All authors have intellectually reviewed and approved the final version of the manuscript. They also agreed to be responsible for all aspects of the work. Detailed substantial contributions were as follows: study concept and design: M.G.‐M., S.S.S. and Mo.Mo.; data collection: M.T. and Mo.Mo.; analysis and interpretation of data: S.S.S. and P.S.S.; drafting of the manuscript and critical revision: P.S.S., Z.F., F.I., Ma.Ma. and G.A.F.

## Ethics Statement

The study protocol was given approval by the Ethics Committee of Mashhad University of Medical Sciences.

## Consent

Written informed consent was obtained from participants.

## Conflicts of Interest

The authors declare no conflicts of interest.

## Data Availability

The data that support the findings of this study are available from Mashhad University of Medical Sciences, but restrictions apply to the availability of these data, which were used under licence for this study, and so are not publicly available. Data are however available from the corresponding author, Majid Ghayour‐Mobarhan (email: ghayourm@mums.ac.ir), upon reasonable request and with permission of Mashhad University of Medical Sciences.
